# A rapid intrinsic heart rate resetting response with thermal acclimation in rainbow trout, *Oncorhynchus mykiss*

**DOI:** 10.1242/jeb.215210

**Published:** 2020-06-15

**Authors:** Rachel L. Sutcliffe, Shaorong Li, Matthew J. H. Gilbert, Patricia M. Schulte, Kristi M. Miller, Anthony P. Farrell

**Affiliations:** 1 Department of Zoology, University of British Columbia, Vancouver, BC, Canada, V6T 1Z4; 2 Pacific Biological Station, Fisheries and Oceans, Nanaimo, BC, Canada, V9T 6N7

**Keywords:** Temperature acclimation, Cardiac, Myogenic heartbeat, Pacemaker, Sinoatrial node, HCN proteins

## Abstract

We examined cardiac pacemaker rate resetting in rainbow trout following a reciprocal temperature transfer. In the original experiment, performed in winter, 4°C-acclimated fish transferred to 12°C reset intrinsic heart rate after just 1 h (from 56.8±1.2 to 50.8±1.5 beats min^−1^); 12°C-acclimated fish transferred to 4°C reset intrinsic heart rate after 8 h (from 33.4±0.7 to 37.7±1.2 beats min^−1^). However, in a replicate experiment, performed in the summer using a different brood year, intrinsic heart rate was not reset, even after 10 weeks at a new temperature. Using this serendipitous opportunity, we compared mRNA expression changes of a suite of proteins in sinoatrial node (SAN), atrial and ventricular tissues after both 1 h and longer than 3 weeks for both experimental acclimation groups to identify those changes only associated with pacemaker rate resetting. Of the changes in mRNA expression occurring after more than 3 weeks of warm acclimation and associated with pacemaker rate resetting, we observed downregulation of *NKA α1c* in the atrium and ventricle, and upregulation of *HCN1* in the ventricle. However, in the SAN there were no mRNA expression changes unique to the fish with pacemaker rate resetting after either 1 h or 3 weeks of warm acclimation. Thus, despite identifying changes in mRNA expression of contractile cardiac tissues, there was an absence of changes in mRNA expression directly involved with the initial, rapid pacemaker rate resetting with warm acclimation. Importantly, pacemaker rate resetting with thermal acclimation does not always occur in rainbow trout.

## Abstract (precis)

**Summary:** Intrinsic heart rate resetting occurs inconsistently with temperature acclimation in rainbow trout; however, it can occur rapidly, within 1 h of warming, without changes in mRNA expression of sinoatrial node proteins.

## INTRODUCTION

In ectothermic animals such as fish, acute increases in temperature result in an increase in metabolic oxygen demand due to a thermodynamic response. Nevertheless, over time, many fish species can acclimate to a new temperature and improve function. Thermal acclimation, as a whole, involves generalized (e.g. homeoviscous adaptation) and tissue-specific changes, probably occurring over different time scales (Fangue et al., 2014; Fu et al., 2018). This study focused on the resetting of the intrinsic cardiac pacemaker rate previously observed in rainbow trout (*Oncorhynchus mykiss*) during thermal acclimation (Aho and Vornanen, 2001; Haverinen and Vornanen, 2007).

**List of abbreviations**

Ct: cycle threshold
ECG: electrocardiogram
*E*^−ΔCt^: expression normalized to reference genes
*E*^−ΔΔCt^: expression normalized to reference genes and inter-run calibrators
FDR: false discovery rate
HCN: hyperpolarization-activated cyclic nucleotide-gated channel
IRC: inter-run calibrator
PCA: principal component analysis
qRT-PCR: quantitative real-time polymerase chain reaction
SAN: sinoatrial node

Intrinsic heart rate represents the myogenic heartbeat without any neural or humoral influences. Thus, intrinsic heart rate can be measured *in vivo* by using pharmacological agents to remove these influences (Ekström et al., 2016; Jose and Taylor, 1969), which are largely cholinergic and adrenergic in fish and vary with temperature (Altimiras and Axelsson, 2004; Ekström et al., 2016; Gilbert et al., 2019; Graham and Farrell, 1989; Jose and Taylor, 1969; Wood et al., 1979). Alternatively, placing the heart in a physiological saline *in vitro*, as we did in the present study, physically removes these influences (Graham and Farrell, 1989; Hiroko et al., 1985).

The myogenic nature of a fish's heartbeat *in vitro* is a direct result of specialized pacemaker cells found in the sinoatrial node (SAN) (Farrell and Smith, 2017; Haverinen and Vornanen, 2007; Newton et al., 2014; Stoyek et al., 2015, 2016). However, the intrinsic rate is not necessarily fixed throughout the life of a fish, especially with respect to temperature. For instance, initial warming typically increases the rate of SAN pacemaker cell depolarizations (Aho and Vornanen, 2001; Haverinen and Vornanen, 2007), driving a faster intrinsic heart rate that reverts immediately on cooling, i.e. an acute response to warming and cooling. However, a longer exposure to a warmer temperature can decrease the rate of SAN pacemaker cell depolarization (Aho and Vornanen, 2001; Haverinen and Vornanen, 2007), driving a slower intrinsic heart rate that does not revert immediately on cooling, i.e. an acclimation response. Thus, resetting of the pacemaker rate with warm acclimation in ectotherms is in the opposite direction to that of the acute warming response, which may then allow for a further acute increase in heart rate before heart rate reaches its maximum at the new acclimation temperature, e.g. with further acute warming or with exercise. Such thermal acclimation of intrinsic heart rate has been studied in a wide range of ectothermic species, including in rainbow trout, goldfish, perch, guppy and medaka, as well as in American bullfrogs, common frogs and wood frogs (Bowler and Tirri, 1990; Harri and Talo, 1975; Hiroko et al., 1985; Lotshaw, 1977).

What is less clear concerning pacemaker rate resetting for rainbow trout is the time scale over which thermal acclimation occurs. It is generally assumed that pacemaker resetting takes several weeks at the new temperature, as reported by Aho and Vornanen (2001). However, Ekström et al. (2016) concluded that pacemaker rate resetting in rainbow trout must occur over longer than 6 weeks because it did not change between the first measurement at 1 day and the last measurement 6 weeks after rainbow trout were moved from 9°C to 16°C. However, alternative unaddressed explanations for their result could be that either pacemaker resetting occurred very rapidly or it may not occur at all under certain conditions.

Therefore, the aims of our study were twofold: (1) to better resolve the time course of pacemaker rate resetting in rainbow trout; and (2) to provide new mechanistic insights into pacemaker rate resetting by measuring gene expression of a suite of proteins potentially involved in the ionic currents associated with cardiac activity in the SAN, atrium and ventricle, given that such studies are quite limited for salmonids (Aho and Vornanen, 2001; Hassinen et al., 2007, 2008, 2017; Haverinen and Vornanen, 2007; Korajoki and Vornanen, 2009, 2012). We used a Fluidigm microfluidics qPCR platform to evaluate mRNA expression of 28 functional proteins in three different cardiac tissues from rainbow trout acclimated to either 4 or 12°C for more than 3 weeks (4–10 weeks). We specifically targeted proteins central to two competing models advanced for cardiac pacemaking in mammals (Lakatta and DiFrancesco, 2009): (1) the calcium clock hypothesis, which suggests that spontaneous depolarization is caused by spontaneous release of calcium sparklets from the sarcoplasmic reticulum via ryanodine receptors; and (2) the membrane clock hypothesis, which suggests that spontaneous depolarization is caused by hyperpolarization-activated cyclic nucleotide-gated (HCN) channels, resulting in a ‘funny’ current. Differences in mRNA expression of functional genes in the SAN, atrium and ventricle were established, and differences associated with thermal acclimation were identified for each tissue type. These characterizations then allowed identification of functional genes with mRNA expression patterns uniquely associated with the demonstrated slowing of pacemaker rate with warm acclimation in the first of our rainbow trout acclimation experiments. However, we unexpectedly found that pacemaker resetting did not occur in the second acclimation experiment. Thus, by comparing gene expression patterns across these two experiments, we could distinguish the changes in mRNA expression potentially associated with pacemaker rate resetting responses during warm acclimation from the more general cardiac responses to warm acclimation.

## MATERIALS AND METHODS

### Study design

The time course of pacemaker rate resetting was followed during both cold acclimation (4°C) and warm acclimation (12°C). We measured intrinsic heart rate after as little as 1 h and as long as 10 weeks following transfer of rainbow trout either from 4°C to 12°C, or from 12°C to 4°C (Table 1). We predicted that pacemaker rate resetting would take longer to occur with cold acclimation than with warm acclimation as a result of basic thermodynamics. The experiments were performed in accordance with the Animal Care Guidelines at the University of British Columbia (UBC) (permit number A15-0035).

**Table 1.**
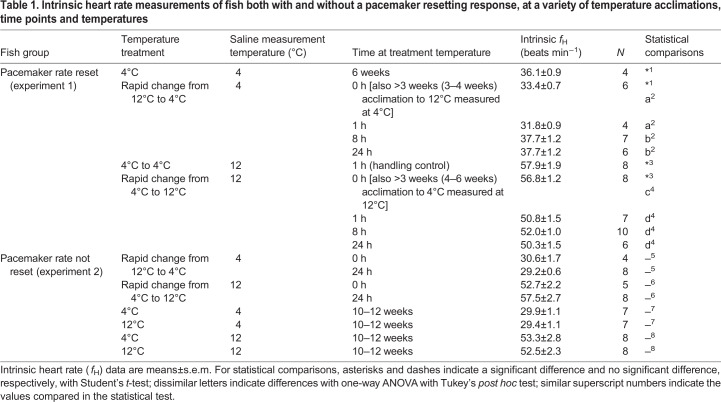
**Intrinsic heart rate** **measurements of fish both with and without a pacemaker resetting response, at a variety of temperature acclimations, time points and temperatures**

### Fish supply, temperature treatments and sampling times

Rainbow trout, *Oncorhynchus mykiss* (Walbaum 1792), were obtained from the Sun Valley Trout Hatchery, where they had been reared in outdoor, flow-through raceways. Two replicate experiments were performed, one over winter from November to December 2016, and a second over early summer from March to June 2017 using a subsequent brood year of trout from the same source. Most of the fish used in experiment 1 were obtained in May 2016 (43 fish) and were held in outdoor flow-through tanks at UBC until October 2016 at a seasonal and unregulated water temperature similar to that at the hatchery. They were then moved into temperature-controlled tanks for the pre-acclimation period. In November 2016, an additional 53 fish from the same brood year were obtained from the same source and added to the pre-acclimation tanks to increase sample sizes. The fish used in experiment 1 had a body mass of 337±0 g (mean±s.e.m.; *n*=96). Fish sex was identified from the gonads when possible; 31% of fish were male (gonad to total body mass ratio of 3.77±0.22%), 33% of the fish were female (gonad to total body mass ratio of 3.90±1.19%) and 36% of fish had undeveloped gonads (gonad to total body mass ratio of 0.34±0.72%). Fish for experiment 2 were placed directly into temperature-controlled pre-acclimation tanks at UBC in March 2017. The fish used in this experiment had a body mass of 258±8 g (*n*=69); 28% of these fish were male (gonad to total body mass ratio of 1.79±0.26%), 36% of the fish were female (gonad to total body mass ratio of 0.97±0.47%) and 36% of fish had underdeveloped gonads (gonad to total body mass ratio of 0.14±0.37%). Consequently, the fish used in the two experiments were the same strain, had similar sex ratios and subsequently received the same experimental treatments. However, they differed in their body mass (*P*<0.0001, Student's *t*-test), brood year and the time of the year that they were acclimated to a constant experimental temperature.

The overall experimental design was the same for the two experiments. First, fish were pre-acclimated to either 4°C (±1°C) or 12°C (±1°C) in recirculating tanks inside an environmental chamber for 3–12 weeks. Daily replacement with temperature-adjusted freshwater ensured good water quality, ammonia levels and oxygen levels (monitored daily). Fish were fed a daily maintenance diet of BioTrout 4.0 mm (Skretting, Vancouver, BC, Canada) and held on a 12 h:12 h photoperiod. A subset of fish was sampled directly from the pre-experimental acclimation tanks (0 h; >3 weeks; 10–12 weeks). Sub-groups of fish were then placed into individual, 19 l mesh-covered buckets and moved into a new experimental tank at the opposite temperature to facilitate the rapid fish sampling needed during the initial stages of thermal acclimation following temperature transfer. Fish were sampled 1, 8 and 24 h at the new acclimation temperature (Fig. S1A). As a control for fish handling, a group of 4°C-acclimated fish were placed in buckets and returned to the 4°C experimental tank and sampled after 1 h. For experiment 1, tissue samples for molecular analysis were taken over 2 days in December from fish returned to 4°C for 1 h after pre-acclimation to 4°C (a fish handling control group) and from fish placed in 12°C for 1 h after their pre-acclimation to 4°C, and from fish acclimated to both 4 and 12°C for >3 weeks (7 weeks and 4 weeks, respectively). Intrinsic heart rate was measured between late November and late December 2016. For experiment 2, tissue samples for molecular analysis were taken during one day in May from fish acclimated to either 4 or 12°C for 10 weeks (referred to as >3 weeks acclimation). Heart rate measurements were taken between early April and early June 2017.

### Protocol to measure intrinsic heart rate

Fish (experiment 1, *n*=66; and experiment 2, *n*=55) were individually killed with a blunt blow to the head, followed by destruction of the brain and spinal cord. The heart, including much of the sinus venosus (to ensure an intact SAN) was quickly excised (<2 min) and was placed, with the ventricle laterally cut to aid the movement of oxygenated (100% oxygen) saline across the heart with each heartbeat, in an organ bath which was held at the appropriate acclimation temperature (either 4±1°C or 12±1°C) by a recirculating chiller. The physiological saline contained 140 mmol l^−1^ NaCl, 2.8 mmol l^−1^ KCl, 1.2 mmol l^−1^ MgSO_4_, 1.8 mmol l^−1^ CaCl_2_, 1.2 mmol l^−1^ NaH_2_PO_4_, 3.9 mmol l^−1^ TES free acid, 6.1 mmol l^−1^ TES Na salt and 10 mmol l^−1^ glucose (pH of 7.8 at 10°C) and its temperature was measured directly. The electrocardiogram (ECG) signal was recorded using two custom-made stainless steel electrodes, one placed next to the SAN and the other away from the heart (Fig. S1B). The ECG signal was amplified (100–1000×) and filtered (high pass: 0.3 Hz, low pass: 0.1 kHz) with a Grass P55 AC amplifier (Astro-Med Inc.). Data acquisition used a PowerLab ML870 and ECG signals were digitally filtered (60 Hz line filter, 0–5 Hz high pass, 45 Hz low pass) and analysed using automated beat detection in LabChart 7 Pro software (ADInstruments). After a stabilization period of 30 min in the saline bath, the stable intrinsic heart rate (beats min^−1^) was recorded as an average from consecutive ECG waveforms (>1 min). Intrinsic heart rate is presented as mean±s.e.m. for *n* individuals. Fish were discarded if either the saline temperature occasionally drifted outside the experimental range after 30 min or heart rate occasionally became arrhythmic or unstable. One-way ANOVA with *post hoc* Tukey’s test and Student’s *t*-test were conducted using Sigmaplot (v. 13.0; Systat Software) and differences were considered significant when *P*<0.05.

### Quantitative reverse transcriptase (qRT-) PCR

#### Cardiac tissue sampling

Hearts were removed as above and placed into oxygenated saline to separate the ventricle (335±20 mg), atrium (58±4 mg) and SAN regions (34±2 mg) (Fig. S1C) for fish (experiment 1, *n*=30; and experiment 2, *n*=14) undergoing temperature acclimation at 4°C or 12°C for >3 weeks, or after 1 h at either 12°C or 4°C following pre-acclimation to 4°C. Each cardiac region was placed into individual Eppendorf tubes for flash freezing with liquid nitrogen. The SAN region can involve the sinoatrial canal (Haverinen and Vornanen, 2007; Newton et al., 2014; Stoyek et al., 2015, 2016) and therefore to ensure inclusion of pacemaker cells, a considerable sample of the sinus venosus tissue was included in the SAN samples along with a minimal amount of atrial tissue. Tissues were stored at −80°C until analysed.

Cardiac genes targeted for qRT-PCR were those potentially implicated in pacemaker function and temperature acclimation (Table S1). All genes had been previously sequenced in rainbow trout (D'Souza et al., 2014; Hassinen et al., 2008; Korajoki and Vornanen, 2009, 2012) with the exception of the *HCN* genes, which have not been previously sequenced. Primer sequences (Table S1) were designed using Primer Express 3 (Life Technologies, Carlsbad, CA, USA) with melting temperatures of 58–60°C (default settings). Specificity of each primer set was tested by cloning and sequencing. Each primer set was run individually on a CFX96 Touch Real-Time PCR Detection System (Bio-Rad, Hercules, CA, USA) with SYBR™ Green PCR Master Mix (Applied Biosystems, Foster City, CA, USA) and a mixed cardiac tissue sample. Cycling conditions were 95°C for 10 min followed by 40 repeats of 95°C for 15 s and 55°C for 1 min. Products were cloned using TOPO™ TA Cloning™ Kit for Subcloning (Invitrogen, Carlsbad, CA, USA), as per the manufacturer's protocol. One Shot™ TOP10 chemically competent *E. coli* cells (Invitrogen) were used for most assays; however, *HCN* isoforms were tested using One Shot™ TOP10F′ *E. coli* cells (Invitrogen) as these produced a higher yield. Plasmid extraction was performed using GeneJET Plasmid Miniprep Kit (Thermo Scientific, Waltham, MA, USA) and the resulting product was sequenced by either the NAPS core facility at UBC (Vancouver, BC, Canada) or Macrogen USA (Rockville, MD, USA). Primers tested produced at least 9–10 clones of the expected sequence and, if not, the primers were discarded. In most cases, apart from isoforms of HCN, we did not try to distinguish between paralogues, unless previously identified in other work. When primer sequences were obtained from previous studies (Table S1), the products of these primers were not cloned and sequenced.

#### RNA extraction

RNA was extracted according to the manufacturer's protocol from all tissue samples using MagMAX™-96 for Microarrays Total RNA Isolation Kit (Ambion, Austin, TX, USA). Briefly, a tissue sample was weighed, and if necessary divided into sections weighing <90 mg. Tissue sections were then placed into 200 µl of TRI reagent^®^ (Sigma-Aldrich, St Louis, MO, USA) and briefly homogenized using a pellet pestle. More TRI reagent^®^ was added to give a final TRI reagent^®^ volume of 15 µl per 1 mg of tissue; 0.2 µl of external standard mRNA (500 pg µl^−1^) per mg of tissue was then added to the tube with roughly ten 1.0 mm ceria-stabilized zirconium oxide beads (Next Advance, Averill Park, NY, USA) in each tube. The tissue samples were fully homogenized with Bullet Blender24 (Next Advance) before adding 0.1 ml of 1-bromo-3-chloropropane per 1 ml of Tri reagent^®^ to each tube and centrifuging at 12,000 ***g*** for 10 min. The aqueous layer was removed and frozen at −80°C for storage. The aqueous layers for tissues weighing >90 mg, which had been processed in sections up till this point, were recombined with volumes relative to section mass, to make up 150 µl [(section mass/total sample mass)×150 µl]. When all samples had been processed to this point, the aqueous layers were defrosted and placed in 96-well plates. RNA extraction then proceeded as in the ‘spin’ protocol using a BioMek FXP (Beckman Coulter, Brea, CA, USA) automated liquid handling instrument. Purity was assessed using *A*_260_/*A*_280_ ratios (1.97–2.45) and yield was calculated using 260 nm absorbance. The RNA concentration was then normalized using the BioMek FXP (Beckman Coulter) automated liquid handling instrument to 62.5 ng µl^−1^.

#### Fluidigm qRT-PCR

qRT-PCR was run using the Fluidigm Biomark™ microfluidics platform (Fluidigm, South San Francisco, CA, USA) with Evagreen^®^ assays as fully described in Jeffries et al. (2014) for 33 target genes and 8 reference genes (Table S1). Briefly, total RNA was used to synthesize cDNA with SuperScript™ VILO™ MasterMix (Invitrogen) as per the manufacturer's protocol. cDNA was pre-amplified in a specific target amplification (STA) step using all the primer pairs (Table S1) and TaqMan™ Preamp Master Mix (Life Technologies). The Biomark protocol for pre-amplification was then followed (1.25 µl of cDNA, 1.25 µl of 200 nmol l^−1^ pooled primer mix and 2.5 µl of Master Mix). Any primers that were unincorporated were then removed using ExoSAP-IT™ High-Throughput PCR Product Clean Up (MJS BioLynx Inc., Brockville, ON, Canada) (2 µl of ExoSAP-IT per 5 µl of post-PCR reaction volume), followed by 1:5 dilution in DNA Suspension Buffer (TEKnova, Hollister, CA, USA).

qRT-PCR was run with 96×96 dynamic arrays using Biomark HD™. A sample premix [2.5 µl 2× SsoFast™ EvaGreen^®^ Supermix with low ROX (Biotium, Fremont, CA, USA), 0.25 µl 20× DNA Binding Dye Sample Loading Reagent (Fluidigm) and 2.25 µl of the diluted pre-amplified product] and an assay premix [2.5 µl of 2× Assay Loading Reagent (Fluidigm), 2.25 µl 1× DNA Suspension Buffer (TEKnova, Hollister) and 50 µmol l^−1^ each of mixed forward and reverse primers] were made. These were loaded onto the 96×96 dynamic array and mixed using an IFC controller HX (Fluidigm). qRT-PCR was then run using the recommended protocol for Evagreen, GE Fast 96×96 PCR+Melt v2 protocol with Biomark HD™ (a thermal mixing protocol of 70°C for 40 min and 60°C for 30 s, then a hot start protocol of 95°C for 60 s, followed by 30 qRT-PCR cycles of 96°C for 5 s and 60°C for 20 s; a melting protocol of 60°C for 3 s followed using a 1°C increase every 3 s up to 95°C).

#### Quality control

A balanced statistical design for ANOVA analyses used 7 samples for each experimental group. mRNA extraction and qPCR were performed on all original 7–10 tissue samples per group and sample sizes were reduced by first excluding any individual where a cardiac tissue type was missing, and then by excluding the last samples that were homogenized.

The Fluidigm approach offers the distinct advantage of simultaneously running many assays, but optimizing conditions so that all assays perform well with differing mRNA expression levels can be difficult. As the aim of this study was only to gain a broad-scale picture, we did not optimize and rerun assays that failed quality criteria, and instead we chose to exclude data for any genes that did not meet our stringent quality control filters*.* Of 33 possible target genes run simultaneously, 28 met our quality control standards and were ultimately used for qRT-PCR analysis [all efficiencies, *r*^2^ and experimental cycle threshold (Ct) ranges are presented in Table S2]. For the target genes we excluded, the standard curves for *α_1A_-adrenoceptor*, *β_2_-adrenoceptor*, *HCN4b* and *NKA α1b* were insufficient, while Ct values for some *ANP* samples were too low for the Fluidigm system (i.e. 2.5Ct) (Table S2). For the remaining 28 target genes, a measurement was excluded from further analysis if the difference between technical replicates was greater than 1 cycle (i.e. *Na_v_1.4* for a single SAN sample from the fish acclimated to 4°C for 1 h with a pacemaker rate resetting response, and *Na_v_1.6* for a single ventricular sample from fish acclimated to 4°C for >3 weeks without a pacemaker rate resetting response).

We utilized data for two reference genes that have been used previously with salmonids (*CCDC84*; Jeffries et al., 2014) and zebrafish (*SEP15*; Xu et al., 2016). Both were confirmed as a suitable combination for an endogenous control using Normfinder (combined stability value of 0.019). Six other reference genes tested (*18s*, *β-actin*, *EF1α*, the external standard, *DnaJA2* and *MprPL40*) were excluded using one of the following strict quality criteria: (a) Ct values were outside the recommended detection limit (i.e. 6–25Ct) for the Fluidigm system (i.e. *18s*, *β-actin*, *EF1α*, *DnaJA2* and *MprPL40*) (Table S2); (b) the efficiency was ≤80% or ≥120% (i.e. *18s* and the external standard, respectively); or (c) *r*^2^ was ≤0.980 (i.e. both *18s* and the external standard). Stricter quality criteria were applied to the reference genes than to the target genes because inaccurate reference gene measurements will affect every assay, whereas inaccurate target gene measurements will only affect that single assay.

Standard curves, run in duplicate on both plates to give quadruplicates, were calculated from serial dilutions (0.2×, 0.04×, 0.008× and 0.0016×) of a cDNA mixture of all samples that was made up before the STA step (Bustin et al., 2009). The undiluted sample (1×) did not have a linear relationship with the other dilutions for any of the assays, and so was not used in the standard curve. When replicates had a range greater than 1Ct, we excluded this dilution. If fewer than three dilutions remained, normally in association with high Ct values, we excluded the whole assay (i.e. *α_1A_-adrenoceptor*, *β_2_-adrenoceptor*, *HCN4b* and *NKA α1b*).

#### Calculating gene expression

##### *E*^−ΔCt^ (normalized quantity)

*E*^−ΔCt^, expression normalized to a housekeeping gene, was calculated for a semi-quantitative comparison of the expression of different target genes. This measure was obtained as in Scott et al. (2004) using the geometric mean of the reference genes as in Hellemans et al. (2007) (Eqn A1).

##### *E*^−ΔΔCt^ (calibrated normalized quantity)

*E*^−ΔΔCt^, expression of the target gene normalized to the expression of the reference genes followed by an inter-run calibrator sample (IRC), a 0.04× dilution of pooled sample (the highest standard curve measurement used), was calculated as in Pfaffl (2001) using the geometric means of the reference genes (Eqn A2).

##### Fold-change in *E*^−ΔΔCt^ with warm acclimation (calibrated normalized relative quantity)

Fold-change in *E*^−ΔΔCt^ of the target gene with warm acclimation was calculated for each pacemaker rate response group, by dividing the *E*^−ΔΔCt12°C^ (Eqn A2) by the *E*^−ΔΔCtaverage4°C^ (Eqn A3), in a method that is mathematically identical to that of Hellemans et al. (2007).

### Statistical analysis of mRNA expression

Principal component analysis (PCA) was performed using the PRCOMP package in R Studio (http://www.R-project.org/) with 68% confidence ellipses used to depict 1 s.d. False discovery rate (FDR) adjustments were performed using GraphPad Prism 7.00 (GraphPad Software) and other statistical analysis was performed using Sigmaplot 14.0 (Systat).

Various comparisons allowed us to examine four primary questions with our qRT-PCR study. First, by comparing the mRNA expression in the SAN, atrium and ventricle in fish acclimated to 4°C for at least 3 weeks (experiment 1) we could ask: how does mRNA expression differ among cardiac tissues? Second, by comparing mRNA expression after acclimation to either 4 or 12°C for at least 3 weeks (experiment 1), we could ask: how does cardiac tissue mRNA expression change with thermal acclimation? Third, by comparing fish in experiments 1 and 2 that had been acclimated for more than 3 weeks to either 4 or 12°C, we could ask: what changes in mRNA expression are specifically associated with pacemaker rate resetting? For these three questions, genes were grouped in three ways. (1) mRNA changes associated with warm acclimation, but not pacemaker rate resetting: for these genes, mRNA expression had to be significantly different between 4 and 12°C in experiments 1 and 2, but the fold-change in mRNA expression not different with warm acclimation between the two experiments. (2) mRNA changes only associated with pacemaker rate resetting: for these genes, mRNA expression had to be significantly different between 4 and 12°C only in experiment 1, in addition to a significant difference in the fold-change in mRNA expression with warm acclimation between the two experiments. (3) mRNA changes associated with no pacemaker rate resetting: for these genes, mRNA expression had to be significantly different between 4 and 12°C, but only in experiment 2, in addition to a significant difference in the fold-change in mRNA expression with warm acclimation between the two experiments.

Lastly, we could ask whether the mRNA expression changes associated with pacemaker rate resetting during full thermal acclimation could also be driving the initial resetting of pacemaker rate by comparing mRNA expression in handling control fish moved from 4°C to 4°C for 1 h, and experimental fish moved from 4 to 12°C for 1 h. The mRNA expression changes seen within this first hour were then compared with those changes in mRNA only associated with pacemaker rate resetting.

#### Comparison of mRNA expression in cardiac tissue types

Differences in mRNA expression patterns were compared between cardiac tissue types with PCA, using *E*^−ΔΔCt^. A 68% confidence limit ellipse was placed around each tissue type to graphically illustrate 1 s.d. for the distribution.

Significant differences in gene expression between cardiac tissues were identified by comparing *E*^−ΔΔCt^ mRNA expression using a one-way ANOVA and a Tukey's *post hoc* test. If the test for either normality (Shapiro–Wilk’s test) or equal variance (Levene's test) failed, a non-parametric Kruskal–Wallis ANOVA and a Dunn's *post hoc* test was used.

For mRNA expression of the membrane clock and calcium clock proteins, relative expression is informative beyond just overall expression. Therefore, pie charts were created to qualitatively compare the relative expression for each tissue type using average *E*^−ΔCt^ values for the mRNA expression of proteins involved in the HCN model and calcium clock model.

A semi-quantitative measure of total mRNA expression for each functional gene group was calculated from the total *E*^−ΔCt^ values of all the genes in each group for each individual. These were compared among cardiac tissues, using a one-way ANOVA and Tukey's *post-hoc* test. If the test for either normality (Shapiro–Wilk’s test) or equal variance (Levene's test) failed, the non-parametric Kruskal–Wallis ANOVA and Dunn's *post hoc* test were used. FDR adjustment was performed within each thermal acclimation group.

#### Comparison of mRNA expression between warm- and cold-acclimated fish

PCA with *E*^−ΔΔCt^ compared differences in mRNA expression patterns for warm- and cold-acclimated fish. A 68% confidence limit ellipse was placed around each thermal acclimation to graphically illustrate 1 s.d. for the distribution.

Significant differences in gene expression between warm- and cold-acclimated fish were identified by comparing *E*^−ΔΔCt^ mRNA expression within tissue and treatment using Student's *t*-test (Fenna, 2013; Hassinen et al., 2008; Korajoki and Vornanen, 2012). If the test for either normality (Shapiro–Wilk’s test) or equal variance (Levene's test) failed, the non-parametric Mann–Whitney test was used.

FDR adjustment was performed within each tissue for each temperature and thermal acclimation group.

#### Comparison of the fold-change in mRNA expression with warming

PCA with the fold-change in *E*^−ΔΔCt^ with warm acclimation compared differences in mRNA expression patterns for fish from experiments 1 and 2. A 68% confidence limit ellipse was placed around each tissue type to graphically illustrate 1 s.d. for the distribution.

Significant differences between the pacemaker rate resetting responses were identified by comparing the fold-change in *E*^−ΔΔCt^ with warm acclimation for mRNA expression between fish from experiments 1 and 2 with Student's *t*-test. If the test for either normality (Shapiro–Wilk’s test) or equal variance (Levene's test) failed, the non-parametric Mann–Whitney test was used. FDR adjustment was performed within each tissue for each thermal acclimation group.

#### Correcting for multiple comparisons

Multiple comparisons increase the likelihood of false positive results. Therefore, FDR adjustments were applied to all ANOVA and Student's *t*-tests using the two-stage linear step-up procedure of Benjamini et al. (2006) with 5% *Q*-values within the groups specified in the methods. Unadjusted *P*-values are also presented for comparison.

### Data presentation

Data, including Venn diagrams for comparisons of cold and warm acclimation and comparisons of the fold-changes in mRNA expression with warm acclimation, were presented using R Studio (http://www.R-project.org/), GraphPad Prism 7.00 (GraphPad Software) and Inkscape 0.92 (Inkscape Team).

## RESULTS

### Pacemaker rate resetting

**Fig. 1.**
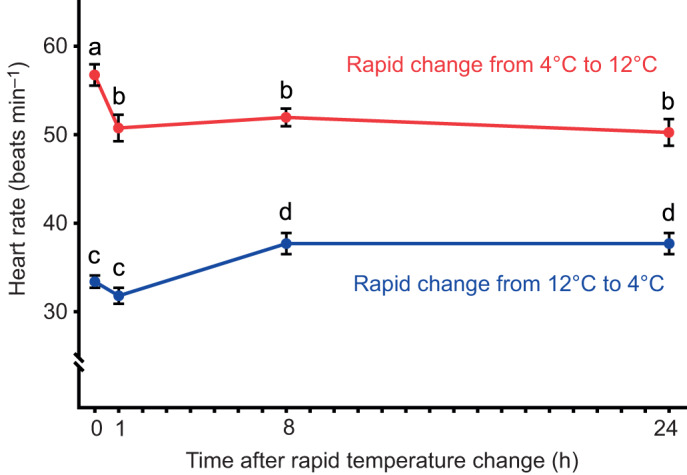
**Changes in intrinsic heart rate** **over time in response to a rapid temperature change.** Data (means±s.e.m.) are for fish rapidly transferred from 4°C to 12°C (experiment 1, performed in winter), with measurements of intrinsic heart rate made at 12°C (*n*=6–9; red), and for fish rapidly transferred from 12°C to 4°C, with measurements of intrinsic heart rate made at 4°C (*n*=4–6; blue). Dissimilar letters indicate statistically significant differences among time points within an experimental group (*P*<0.05, one-way ANOVA).

In experiment 1, resetting of intrinsic heart rate occurred rapidly with both warm acclimation and cold acclimation (Fig. 1). When measured at 4°C, intrinsic heart rate was significantly higher after 4–6 weeks in 4°C-acclimated fish versus 12°C-acclimated fish (*P*=0.044, Student's *t*-test) (Table 1 – statistical test 1). After transferring 4°C-acclimated fish to 12°C, pacemaker rate resetting was first seen after 1 h, and the new rate persisted through to the 24 h measurement (Fig. 1, Table 1 – statistical test 4). In contrast, control fish transferred back to 4°C maintained the same intrinsic rate beyond 1 h (Table 1 – statistical test 3), confirming that the temperature change had triggered the resetting of the pacemaker rate. Similarly, the pacemaker rate was reset when 12°C-acclimated fish were moved to 4°C, but 8 h after transfer rather than 1 h. Again, the resetting persisted to the 24 h measurement (Fig. 1, Table 1 – statistical test 2).

In experiment 2, neither temperature acclimation triggered resetting of the pacemaker rate. When 4°C-acclimated fish were moved to 12°C, intrinsic heart rate was unchanged after 24 h and 10–12 weeks (Table 1 – statistical tests 6 and 8). Similarly, when 12°C-acclimated fish were moved to 4°C, intrinsic heart rate was unchanged after 24 h and 10–12 weeks (Table 1 – statistical tests 5 and 7)*.*

### mRNA expression

#### Differences in mRNA expression among tissues for fish acclimated to 4°C for >3 weeks

**Fig. 2.**
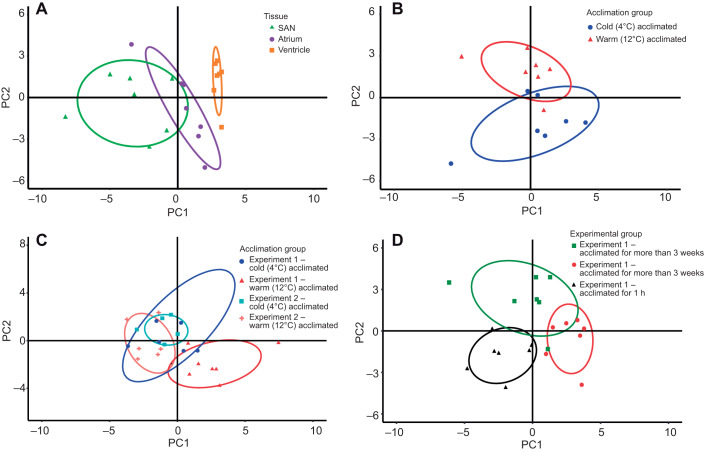
**Principal component analysis (PCA) of mRNA expression of cardiac function genes.** (A) A comparison of tissue types. All *E^−^*^ΔΔCt^ (expression normalized to reference genes and then to an inter-run calibrator, IRC) data shown in Fig. 3 were used in this PCA analysis for sinoatrial node (SAN), atrial and ventricular mRNA expression for fish acclimated to 4°C for more than 3 weeks in experiment 1 (*n*=7). PC1 and PC2 explained 35.9% and 19.4% of variance, respectively. Each tissue type is identified by a different symbol and a 68% confidence limit ellipse. (B) A comparison of the two acclimation temperatures for SAN tissue. All *E^−^*^ΔΔCt^ shown in Fig. S4A were used in this PCA analysis of the SAN of fish acclimated to 4 and 12°C in experiment 1 (*n*=7). PC1 and PC2 explained 24.0% and 22.1% of variance, respectively. Each acclimation group is identified by a different symbol and a 68% confidence limit ellipse. (C) A comparison of the two acclimation temperatures for SAN tissue in fish from experiments 1 and 2. All *E^−^*^ΔΔCt^ shown in Fig. S4A,D were used in this PCA analysis of the SAN of fish acclimated to 4 and 12°C in experiment 1 (*n*=7). PC1 and PC2 explained 23.60% and 17.6% of variance, respectively. Each acclimation group is identified by a different symbol and a 68% confidence limit ellipse. (D) A comparison of the changes in mRNA expression with warming in experiments 1 and 2, for more than 3 weeks and 1 h, for SAN tissue. All fold-changes in *E^−^*^ΔΔCt^ shown in Fig. S5A and similar data for fish from experiment 1 acclimated for 1 h were used in this PCA analysis of the SAN for fish acclimated to 4 and 12°C in experiment 1 (*n*=7). PC1 and PC2 explained 27.4% and 19.7% of variance, respectively. Each experimental group is identified by a different symbol and a 68% confidence limit ellipse. Component loadings are given in Table S3.

**Fig. 3.**
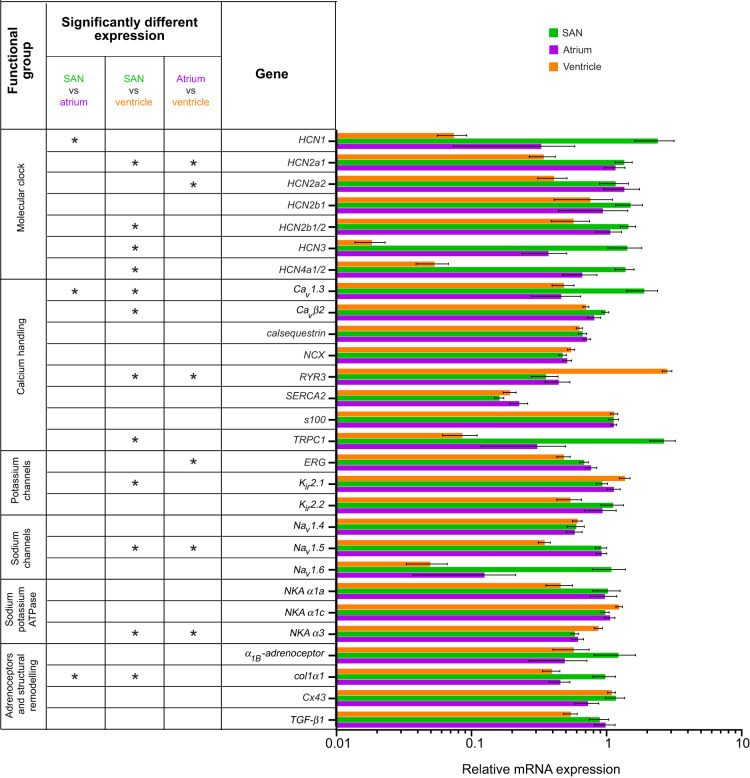
**mRNA expression of cardiac function genes in SAN, atrial and ventricular tissue from fish acclimated to 4°C for more than 3 weeks (experiment 1).** Expression (*E*^−ΔΔCt^) was normalized to the geometric mean of the expression of reference genes (*CCDC84* and *SEP15*) and then to an inter-run calibrator, and values are presented as means±s.e.m. (*n*=7). Statistical comparisons among cardiac tissues are shown on the left for the gene expression data displayed on the right. *Statistically significant differences after FDR adjustment (*P*<0.05).

PCA revealed similar mRNA expression differences among tissues in experiments 1 and 2 (Fig. S2). Focusing on experiment 1, PCA analysis revealed mRNA expression in the ventricle was distinct from that in both the SAN and the atrium (Fig. 2A)*.* PC1 and PC2 explained 35.9% and 19.4% of variance in gene expression, respectively, with a minor overlap of the 68% confidence intervals for PC1 for the SAN and the atrium (Fig. 2A), perhaps reflecting the minor atrial tissue content of the SAN sample. Nevertheless, mRNA expression in the SAN and atrium differed specifically for the membrane clock component *HCN1*, the calcium clock component *Ca_v_1.3* and *col1α1* (Fig. 3). Furthermore, the mRNA expression of membrane clock proteins was higher for SAN than for atrial tissue (Fig. S3A), unlike the calcium handling protein mRNA expression (Fig. S3B) which was similar among the three cardiac tissues.

#### mRNA expression changes as a result of warm acclimation

PCA revealed differences in mRNA expression for warm and cold acclimation in the SAN of fish from experiment 1 (Fig. 2B). PC1 and PC2 explained 24.0% and 22.1% of variance in gene expression, respectively. Pacemaker rate resetting with warm acclimation in experiment 1 was associated with the differential expression of 20 genes: 6 genes in the SAN, 5 genes in the atrium and 9 genes in the ventricle (Table 2). Specifically, a membrane clock component (*HCN2a2*) was significantly downregulated with warm acclimation in the SAN in association with a lower intrinsic heart rate, but not in the atrium (Table 2)*.* Notably, *HCN4a1/2* was the dominant HCN isoform, but it was upregulated relative to total *HCN* expression in all three cardiac tissues (Fig. S3A)*.* Warm acclimation also downregulated mRNA expression of calcium clock components *calsequestrin* and *SERCA2* in both the SAN and atrial tissues, while mRNA expression of *S100* was downregulated only in the SAN and mRNA expression of *RYR3* was downregulated only in the atrium (Table 2)*.* mRNA expression of *NKA α1a* and *NKA α1c* was also downregulated in both the SAN and the atrium. However, mRNA expression of *K_ir_2.1* and *K_ir_2.2*, *ERG*, *Cx43* and *col1α1* was unchanged in both the atrium and the ventricle. While *SERCA2* was downregulated in the ventricle of fish that reset pacemaker rate, calsequestrin was not (Table 2)*.*

**Table 2.**
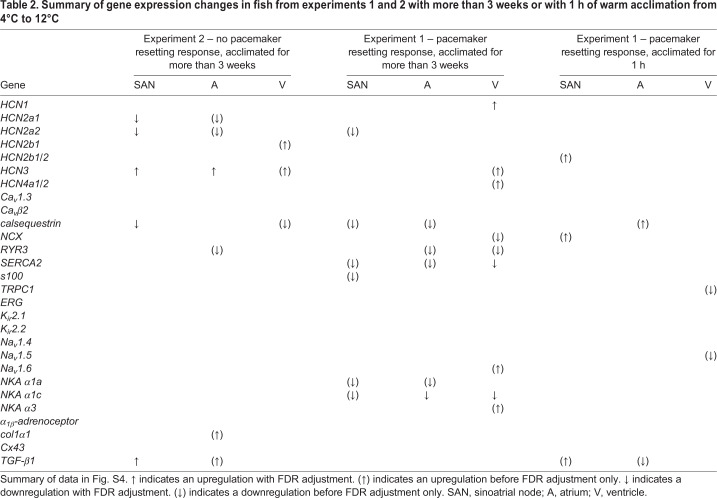
**Summary of gene expression changes in fish from experiments 1 and 2 with more than 3 weeks or with 1 h of warm acclimation from 4°C to 12°C**

#### mRNA expression changes associated with pacemaker rate resetting

PCA revealed that the differences in mRNA expression in the SAN for warm and cold acclimation seen in experiment 1 after more than 3 weeks were not seen in experiment 2 (Fig. 2C). PC1 and PC2 explained 23.6% and 17.6% of variance in gene expression, respectively, but the 68% confidence intervals for both cold- and warm-acclimated fish from experiment 2, and cold-acclimated fish from experiment 1 covered similar areas; only the 68% confidence interval for warm-acclimated fish from experiment 1 showed any separation with only slight overlaps.

PCA also revealed differences in the fold-change of mRNA expression with warm acclimation in fish from experiment 1 acclimated for 3 weeks and for 1 h, and in fish from experiment 1 acclimated for 3 weeks. PC1 and PC2 explained 27.4% and 19.7% of variance in the fold-change in gene expression with warm acclimation in the SAN, respectively (Fig. 2D), with only minor overlaps in the confidence intervals between the three experimental groups.

**Fig. 4.**
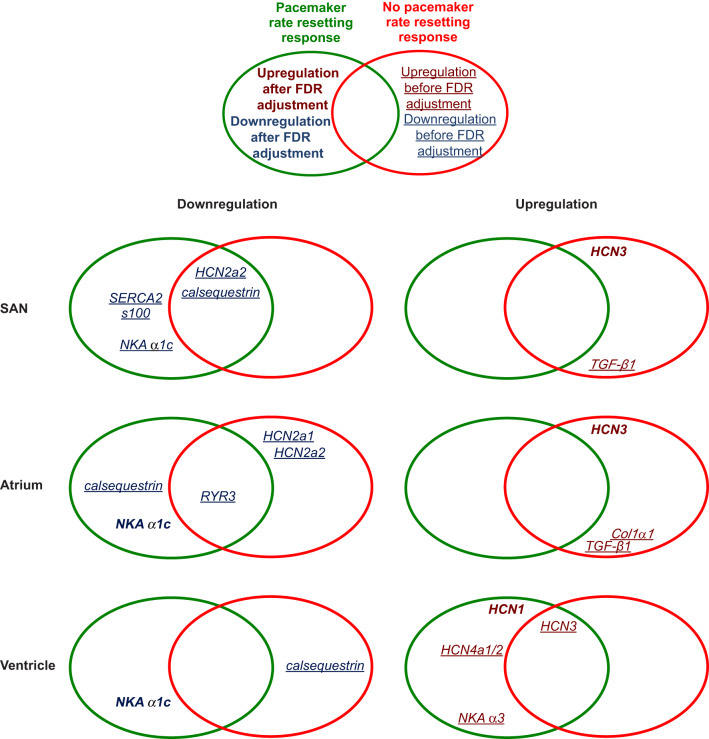
**Significant changes in mRNA** **expression after more than 3 weeks of warm acclimation.** Venn diagrams show changes that were only present in fish that either reset intrinsic heart rate (experiment 1) or did not reset intrinsic heart rate (experiment 2), as well as changes that occurred in both of these groups of fish. The full dataset is shown in Figs S4 and S5. Genes in the green ellipses only are those for which mRNA expression changed only in association with pacemaker rate resetting, i.e. the change was significantly different at 4 and 12°C only in experiment 1, and the fold-change in mRNA expression with warm acclimation was significantly different between the two experiments. Genes in the red ellipses only are those for which mRNA expression changed only in association with no pacemaker rate resetting, i.e. the change was significantly different at 4 and 12°C only in experiment 2, and the fold-change in mRNA expression with warm acclimation was significantly between the two experiments. Genes within both a green and a red ellipse are those for which mRNA expression changed only in association with warm acclimation, but not pacemaker rate resetting, i.e. the change in mRNA expression was significantly different at 4 and 12°C in experiment 1 and 2, and the fold-change in mRNA expression with warm acclimation was not significantly between the two experiments. Genes in a blue font were downregulated and genes in a red font were upregulated. Underlined genes only showed changes in expression before FDR adjustment, while bold genes showed changes in expression after FDR adjustment.

Of the 20 significant changes in gene expression associated with warm acclimation, 9 genes fitted our criteria for ‘mRNA changes only associated with pacemaker rate resetting’ (i.e. they were observed only in experiment 1) (Fig. 4). Specifically, downregulation of calcium clock components *S100* and *SERCA2* mRNA expression occurred only in SAN tissue, downregulation of *calsequestrin* mRNA expression occurred only in atrial tissue, and upregulation of *HCN1*, *HCN4a1/2* and *NKA α3* mRNA expression occurred only in ventricular tissue specific to the fish that reset pacemaker rate. Downregulation of *NKA α1c* expression with warm acclimation was the only change common to the SAN, atrial and ventricular tissues and unique to the fish in experiment 1 (Table 2; Fig. S5). However, after FDR adjustment, only the upregulation of *HCN1* in the ventricle and the downregulation of *NKA α1c* in the atrium and ventricle remained significant (Fig. 4).

Of the 14 significant changes in gene expression associated with warm acclimation in experiment 2 (5 in the SAN, 6 in the atrium and 3 in the ventricle), 6 genes fitted the criteria for ‘mRNA changes associated with no pacemaker rate resetting’ (i.e. they were observed only in experiment 2) ( Fig. 4)*.* However, after FDR adjustment, only the upregulation of membrane clock component *HCN3* in SAN and atrial tissues remained significant (Fig. 4).

Of the remaining gene expression changes, 4 genes fitted the criteria for ‘mRNA changes associated with warm acclimation, but not pacemaker rate resetting’ (Fig. 4)*.* However, after FDR adjustment, none of these changes remained significant (Fig. 4).

While some mRNA expression changes were observed 1 h after warm acclimation in fish with a pacemaker rate resetting response (Table 2), none of these changes were significant after FDR adjustment. In addition, none of these genes showed expression patterns that fitted the criteria for ‘mRNA changes only associated with pacemaker rate resetting’ in the fish acclimated for >3 weeks (Table 2, Fig. 4).

## DISCUSSION

Before our study was undertaken, it was assumed that differences observed in the occurrence of the pacemaker resetting response with thermal acclimation were either species specific (Matikainen and Vornanen, 1992) or due to an insufficient temperature acclimation period (Ekström et al., 2016). However, unexpected results from our study demonstrated that the occurrence of pacemaker resetting with thermal acclimation is not always assured in rainbow trout. While experiment 1 revealed the expected intrinsic heart rate resetting in response to both warm and cold acclimation, experiment 2 did not show intrinsic heart rate resetting, even after 10 weeks acclimation to either 4 or 12°C. This means that the lack of pacemaker resetting responses in fishes cannot be attributed solely to species differences, as suggested previously (Matikainen and Vornanen, 1992). Indeed, differences in both intrinsic heart rate and the electrophysiology of cardiac myocytes have been previously observed in a number of fish species, although not in salmonids (Filatova et al., 2019; Harri and Talo, 1975; Matikainen and Vornanen, 1992). What might turn off pacemaker resetting awaits further study, but the present results suggest seasonal acclimatization could be a factor, given that the two experiments were performed at different times of the year. Previous life experiences could also be a factor as work in our own laboratory has shown that a severe hypoxic stress event could be a possible stimulus for a lack of intrinsic heart rate resetting (Sutcliffe, 2018) and our two experimental groups were from different brood years with different life experiences.

The second novel discovery from our study was the speed at which intrinsic heart rate resetting can occur. By reciprocally transferring rainbow trout to either 4 or 12°C in experiment 1, intrinsic heart rate became significantly different after just 1 h at 12°C and after 8 h at 4°C. The changes in heart rate were in the direction expected, adding confidence to our novel finding of rapid intrinsic heart rate resetting. Furthermore, the change in intrinsic heart rate occurred faster with warm acclimation, as expected, and most likely as a result of faster reaction rates at higher temperatures. Changes in critical thermal maximum and minimum (CT_max_ and CT_min_) in sheepshead minnow similarly occur more rapidly with warm acclimation versus cold acclimation (Fangue et al., 2014).

Few studies have previously examined the time scale for intrinsic heart rate resetting during temperature acclimation; instead, heart rate has typically been measured after many days or weeks. For example, heart rate resetting was observed after 4–10 days in American bullfrogs and wood frogs (Lotshaw, 1977), after 2 weeks in the common frog (Harri and Talo, 1975), after 3 weeks in perch (Talo and Tirri, 1991), after 4 weeks in rainbow trout (Aho and Vornanen, 2001) and after 2 months in goldfish (Hiroko et al., 1985; Tsukuda, 1990). Only Ekström et al. (2016) designed an experiment to examine the time course of thermal acclimation of heart rate; they followed rainbow trout from 1 day to 6 weeks after transfer from 9°C to 16°C. However, because they observed no intrinsic heart rate resetting, they concluded that pacemaker rate resetting takes longer than 6 weeks. However, our study supports two alternative explanations: (1) intrinsic heart rate may not reset at all in some rainbow trout populations or conditions, as in our experiment 2; or (2) the resetting had been completed before their first measurement at 24 h, as in our experiment 1. Whatever the explanation, our study demonstrates that pacemaker resetting can occur at a significantly faster rate than previously assumed, within hours rather than weeks, even with cold acclimation.

Pacemaker resetting is not the only cardiac response to thermal acclimation. While not the focus of our study, other studies of cardiac thermal acclimation responses have identified changes in the stiffness of the cardiac tissues, in the density of cardiac β-adrenergic receptors and in the cardiac sensitivity to β-adrenergic receptor stimulation (Aho and Vornanen, 1999, 2001; Altimiras and Axelsson, 2004; Anttila et al., 2013; Brett, 1971; Casselman et al., 2012; Chen et al., 2013, 2015; Costa et al., 2002; Drost et al., 2014; Eliason et al., 2011, 2013; Farrell, 1991; Fry, 1947; Gilbert et al., 2019; Graham and Farrell, 1989; Keen et al., 1993, 2016, 2017; Klaiman et al., 2011, 2014; Shiels and Farrell, 1997; Steinhausen et al., 2008; Verhille et al., 2013; Wood et al., 1979). Whether or not these responses are homogeneous with thermal acclimation, or even occurred in our study is unknown. However, previous studies have linked changes in mRNA expression in atrial and ventricular tissues with temperature acclimation and some of these changes were observed in our study. For example, and similar to Hassinen et al. (2007) and Korajoki and Vornanen (2012, 2009), we observed that fish with an intrinsic heart rate resetting response had no change in *K_ir_2.1* and *K_ir_2.2* expression, a downregulation in *SERCA2* in atrial and ventricular tissues, and a downregulation of *calsequestrin* in atrial but not ventricular tissue during warm acclimation. However, other previously identified differences in mRNA expression with warm acclimation, i.e. *ERG*, *Cx43* and *col1α1* (Fenna, 2013; Hassinen et al., 2008), were not observed in the present study. Whether these differences reflect different experimental temperatures among studies (4 and 12°C in our study versus 4 and 18–19°C previously) or some other variable is unclear. While previous studies report other cardiac changes during thermal acclimation, heart rate was not measured in parallel with mRNA expression; therefore, we cannot be sure whether these previous studies involved fish with or without an intrinsic heart rate resetting response.

SAN tissue, rather than ventricular or atrial tissue, is responsible for resetting pacemaker rate. Therefore, mRNA expression differences between SAN tissue and two other cardiac tissues probably reflect a role in pacemaking. However, as pacemaker cells occupy a very small region of the heart and cannot be easily defined by eye, we conservatively dissected the SAN tissue to reduce the risk of accidently missing SAN tissue, but this meant that a small portion of atrial tissue was included in the SAN tissue sample. Despite this potential contamination, mRNA expression patterns seen in the SAN tissue were not necessarily the same as those seen in the atrial tissue, and vice versa. For example, *HCN1*, *col1α1* and *Ca_v_1.3* were differentially expressed between the atrial and SAN tissues. Therefore, the mRNA expression ascribed solely to the SAN tissue is done with some confidence and, if anything, any expression differences identified would potentially be underestimated.

Our study is the first in fish to examine the mRNA expression of components of the calcium and the membrane clock in SAN tissue. Both clocks are hypothesized to drive spontaneous depolarization of pacemaker cells in the SAN in mammals, and evidence exists that this may (Hassinen et al., 2017; Marchant and Farrell, 2019) or may not be the case in fish (Wilson and Farrell, 2013). Therefore, the differentially higher expression of *HCN1*, a membrane clock component, observed in the SAN compared with the atrial tissue is of significant interest. This is similar to observations in mammals (Marionneau et al., 2005) where HCN1 is the fastest activating HCN isoform and is activated at the highest voltage (Wahl-Schott and Biel, 2009). If rainbow trout HCN1 has similar properties, then its enhanced expression in the SAN tissue could mean that the membrane clock is being modulated to alter the intrinsic pacemaker rate. This, along with physiological experiments demonstrating that HCN antagonists can slow down *in vivo* heart rate in rainbow trout (Altimiras and Axelsson, 2004; Keen and Gamperl, 2012), and that calcium antagonists have only small effects on heart rate (Haverinen and Vornanen, 2007), lends support to the membrane clock hypothesis for spontaneous depolarization in rainbow trout. However, a high *HCN1* mRNA expression need not necessarily correlate with a functional role in the spontaneous depolarization of pacemaker cells. Indeed, the HCN4 isoform dominates in all cardiac tissues from rainbow trout. Furthermore, in brown trout, cardiac pacemaker cells have a high HCN expression but no *I*_f_ (‘funny’ or pacemaker) current, suggesting a membrane clock may not function at all in the spontaneous depolarization of pacemaker cells in this species despite HCN expression (Hassinen et al., 2017). Thus, further work is needed to resolve the mechanism responsible for spontaneous depolarization in salmonids, and further studies of mRNA expression may not be the best way forward.

Key to understanding what drives pacemaker rate is understanding what causes it to reset. Despite this, changes in mRNA expression with temperature acclimation have not been previously examined in the SAN tissue of any fish. If *HCN1* and other membrane clock components set the pacemaker rate, we would expect changes in expression of these isoforms in the SAN after fish reset intrinsic heart rate with warm acclimation. However, we observed very few changes in mRNA expression that were unique to the SAN. These were restricted to downregulation of *HCN2a2* and *s100*, components of the membrane clock and the calcium clock, respectively. However, if a change in mRNA expression is associated with pacemaker rate resetting, it will probably not only be observed in fish with a pacemaker rate resetting response but also be unique to those fish. Identifying these changes was greatly aided by comparisons between fish in experiments 1 and 2 to identify genes with a significantly different mRNA expression between warm- and cold-acclimated fish, but only in fish with a pacemaker rate resetting response and with a greater fold-change in mRNA expression with warm acclimation. The genes most likely to be responsible for these changes are those which were expressed in the SAN. However, no mRNA expression changes unique to the fish that reset intrinsic heart rate were significant after FDR adjustment. Therefore, we did not identify strong links between changes in mRNA expression and intrinsic heart rate resetting.

This leads back to the rapid pacemaker resetting response observed *in vitro.* None of the mRNA expression changes that were unique to the fish that reset intrinsic heart rate with >3 weeks acclimation were present after just 1 h of warm acclimation. While changes in mRNA expression might not be expected to occur so rapidly, this lack of association does suggest that the initial and rapid resetting of pacemaker rate, and perhaps pacemaker resetting in general, is not driven by changes in mRNA expression. Therefore, future studies of pacemaker setting in fish should consider alternative mechanisms, such as post-transcriptional protein modification.

In conclusion, pacemaker rate resetting with thermal acclimation occurred much more rapidly in the present study than previously shown in any ectotherm, even though we also showed that the pacemaker rate does not always reset with thermal acclimation in rainbow trout. The rapid pacemaker rate resetting response, however, was not associated with any significant changes in mRNA expression for key proteins in the SAN, and we did not identify any mRNA expression changes that would be a strong candidate to be driving the pacemaker resetting response. Nevertheless, differential changes in mRNA expression during thermal acclimation were discovered among SAN, atrial and ventricular tissues, including novel candidate genes in SAN tissue that are associated with warm acclimation but independent of resetting pacemaker rate.

## Supplementary Material

Supplementary information

## APPENDIX

*E*^−ΔCt^, gene expression normalized to that of a reference gene (here *CCDC84* and *SEP15*), was used to qualitatively compare relative differences in gene expression between genes and tissue types. This was calculated as:

(A1)

*E*^−ΔΔCt^, expression of the target gene normalized to the expression of the reference genes and then to an inter-run calibrator (IRC), was used to compare differences in gene expression between tissue types and between warm- and cold-acclimated fish. This was calculated as:

(A2)

Fold-change in *E*^−ΔΔCt^ of the target gene with warm acclimation was used to compare differences in mRNA expression patterns with warm acclimation for fish from experiments 1 and 2. This was calculated by dividing the *E*^−ΔΔCt12°C^ (Eqn A2) by the *E*^−ΔΔCtaverage4°C^ (Eqn A3):

(A3)
