# Supplementary material for: A rapid intrinsic heart rate resetting response with thermal acclimation in rainbow trout, Oncorhynchus mykiss
Source: J Exp Biol. 2020 Jun 15;223(12):jeb215210. doi: 10.1242/jeb.215210 (PMC7328139; doi:10.1242/jeb.215210)
Supplement: Supplementary information [file jexbio-223-215210-s1.pdf]

Supplementary Information

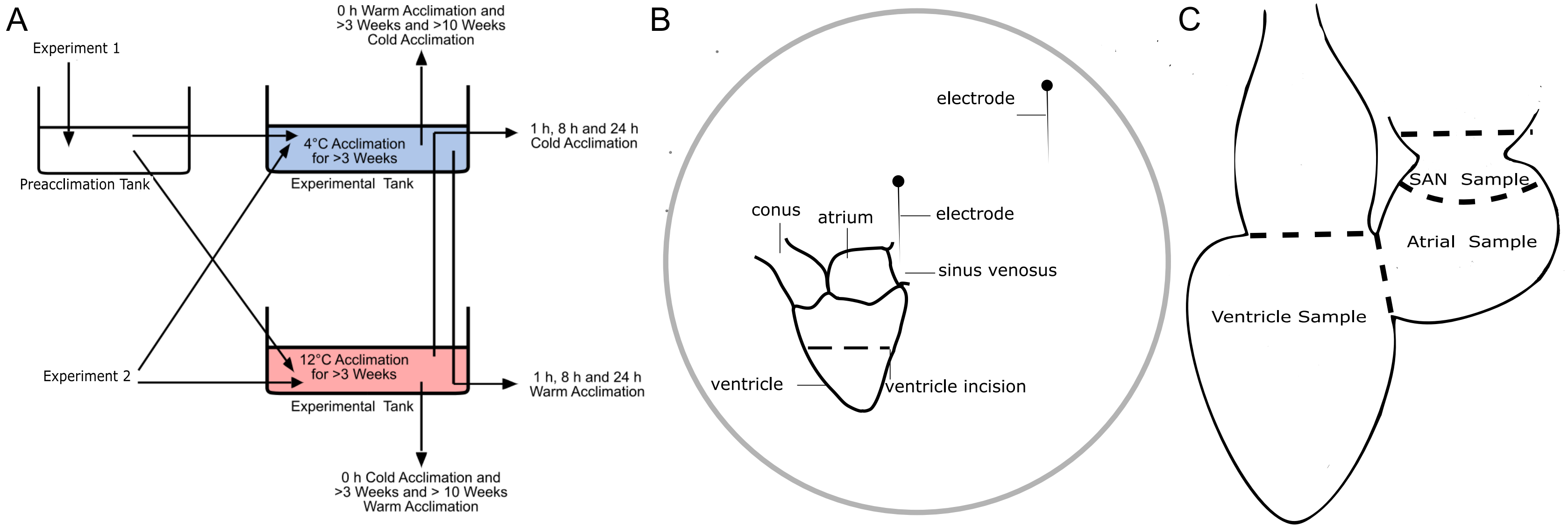

Fig. S1. Experimental protocol schematics. (A) A schematic of the experimental design used to follow the time course of warm and cold acclimation in rainbow trout (B) A diagram of the experimental set up for *in vitro* heart rate measurement (C) A diagram of the cardiac tissue sections used for ventricle, atrial and SAN-containing sample.

Journal of Experimental Biology • Supplementary information

Table S1. Primer pairs used for Fluidigm qRT-PCR analysis.

Target	Forward Primer	Reverse Primer	Accession Number/ Source Study
18s (Reference Gene)	CGGTCGGCGTCCAACTT	CAATCTCGCGTGGCTGAA	AF243428
β-actin (Reference Gene)	TGGGGCAGTATGGCTTGTA TG	CTGGCACCCTAATCACTCT	Ojima (2007)
CCDC84 (Reference Gene)	GCTCATTTGAGGAGAAGGA GGATG	CTGGCGATGCTG TTCCTGAG	Jeffries et al. (2014)
DnaJA2 (Reference Gene)	TTGTAATGGAGAAGGTGAG G	TGGGCCGCTCTCTTGTAT GT	Hassinen et al. (2007)
EF1-α (Reference Gene)	ACCCTCCTCTTGGTCGTTT	TGATGACACCAACAGCAA CA	Raida and Buchmann (2007)
External standard (Reference Gene)	GTGCTGACCATCCGAG	GCTTGTCCGGTATAACT	Ellefsen et al. (2008)
Mrpl40 (Reference Gene)	CCCAGTATGAGGCACCTGA AGG	GTTAATGCTGCCACCCTCT CAC	Jeffries et al. (2014)
SEP15 (Reference Gene)	TCACAGCAAACCACATTTTG G	AAGATGCCCAGAGTGACA CACA	AY255833
Hyperpolarisation-activated cyclic nucleotide-gated channel 1 (HCN1)	CGCTGAGGATCGTGAGGTT T	TGAGCCGGGAAAGTCTCA GT	AF421883
Hyperpolarisation-activated cyclic nucleotide-gated channel 2a1 (HCN2a1)	ATCGTGGACTTTGTCTCCTC CAT	GATCCCCTTCTCCACGATC A	XM_014141001
Hyperpolarisation-activated cyclic nucleotide-gated channel 2a2 (HCN2a2)	CTGCAGGACTTCCCCTCAG A	CCAGGTGTCATTCACCATC TTG	XM_014191309

Journal of Experimental Biology • Supplementary information

Hyperpolarisation-activated cyclic nucleotide-gated channel 2b1 (HCN2b1)	CGCCAGTACCAGGAGAAGTACA	AGCTGGCAGTTTGTGGAAAGA	XM_014148806
Hyperpolarisation-activated cyclic nucleotide-gated channel 2b1/2 (HCN2b1/2)	CCAGTGCAGTGATGCGTATCTT	ACAGCCATCCCAGTGACACA	AY148882
Hyperpolarisation-activated cyclic nucleotide-gated channel 3 (HCN3)	ACGGACGTATGGCTGACTATCA	CCGGAAACATGGCATAGCA	XM_014176849
Hyperpolarisation-activated cyclic nucleotide-gated channel 4a1/2 (HCN4a1/2)	TGGGAGGAGATCTTCCATATGAC	CAGGTTGACGATACGCACCAT	XM_014175391 and XM_014126754.1
Hyperpolarisation-activated cyclic nucleotide-gated channel 4b (HCN4b)	CGGGCGCTGAGAATCGT	CGGAGCAACCTCAACAAACTC	XM_014170228
L-type calcium voltage-gated channels subunit α1D (Ca_v1.3)	CGGCAAGTCGCCCAAGT	GCGGAGCGTGCTCGTAGTAG	NM_001124328
L-type calcium voltage-gated channels subunit β2 (Ca_vβ2)	TGACATAGATGCCACAGGCTTAGA	GAGCGGAGGTGGACTGGAA	DQ198264
Calsequestrin	CCAACCCTACATCAAATTCTTTGC	TCATTTTCAGGGTCAGCTCCTT	NM_001160499
Sodium-calcium exchanger (NCX)	GCAATGCCGTCAACGTCTT	GGTAGATGGCAGCGATGGA	NM_001124598
Ryanodine receptor 3 (RYR3)	AGGCTTCCTCGGCTTTCAC	TGTCGGAAGTTGGAGATCTTCTT	EF032937
Sarcoplasmic/ endoplasmic reticulum calcium ATPase 2 (SERCA2)	GTGCTCGTCACGATAGAGATGTG	CAGCAGGGACTGGTTCTCTGA	Primers designed off sequences from Korajoki and Vornanen (2012)
S100 calcium binding protein (S100)	GTCAAGACTGGAGGCTCAGAG	GATCAAGCCCCAGAAGTGTTTG	Jeffries et al., 2014

Journal of Experimental Biology • Supplementary information

Transient receptor potential cation channel subfamily C member 1 (TRPC1)	GAAGCGGAAGCGTGATGAG	GGTAGCGGTGGACAAGACAAC	NM_001185053
Ether-à-go-go-related gene (ERG)	TCCACGACGCACGAGAAAC	ACACGACTGGTCTGGAAATGAGT	NM_001124676
Potassium voltage-gated channel subfamily J member 2 (K_{ir}2.1)	GCCCCAGAGCCGCTTT	AGAGACATTGATGAACTGCACGTT	DQ435674
Potassium voltage-gated channel subfamily J member 12 (K_{ir}2.2)	CTACGGCTACCGCTGTGTGA	GGACTGGAAGACCACCATGAA	DQ435676
Sodium voltage-gated channel α subunit 4 (Na_v1.4)	TGTGCTCCGAGCCCTTAAA	CGCCCACAATGGTTTTCAG	EF203231
Sodium voltage-gated channel α subunit 5 (Na_v1.5)	TGCCACCCCTGCTGGTA	CGTGGACTGCTTTCCCAGAT	EF203232
Sodium voltage-gated channel α subunit 8 (Na_v1.6)	CGACACCTTCACCTGCAACA	CATTGGTGACGAAGTCGTTCA	EF203233
Sodium/potassium ATPase subunit α1a (NKA α1a)	CCTTGGATGAGCTTAACAGGAAA	GCCCGAACCGAGGATAGAC	AY319391
Sodium/potassium ATPase subunit α1b (NKA α1b)	CCCATGGATTTGCTGGGTAT	CCTCCATGTCGTTCATTATCTTGT	AY319390
Sodium/potassium ATPase subunit α1c (NKA α1c)	TGCCAGAGGTATTGTCATCAACAC	GAGGCGAGGGTAGCAATACG	AY319389
Sodium/potassium ATPase subunit α3 (NKA α3)	GCCAGCAATGGACATATGAACA	ACTGACGAAGAAGGCTGTGTGA	AY319388
a_{1A}-adrenoceptor	TGGGCTCTGTCTGTCACAATCT	AGGCGCCGGCTCCTT	NM_001124653

Journal of Experimental Biology • Supplementary information

a_{1B}-adrenoceptor	ACCGAAGAACCGGGCTATG	CATAAGCGGCAGGTAGAAGGA	NM_001124650
β_2-adrenoceptor	GCCTAAGCCCCAAGGACAAG	AGCTCCACGGTCCCAACAT	NM_001124440
ANP precursor	TCTGCTCCTGCTTTGTCAACA	AGGGTATGGTCTGCCCAACA	NM_001124211
Collagen type 1a (col1α1)	CCCGAGCCATGCCAGAT	CAGATAACTTCGTCGCACATGAC	NM_001124177
Connexin 43 (Cx43)	TGGCAGCACCATCTCCAA	GGGTGTCGTCAGGGAAGTCA	NM_001124563
Transforming growth factor β1 (TGF-β1)	GGGCTGGAAGTGGATCCAT	GGGCCGATGCAGTAGTTAGC	KF870471

All primers were designed specifically for this study from the sequence given by the accession number or copied directly from the study, unless indicated otherwise. Primers are displayed in the 5' to 3' direction.

Journal of Experimental Biology • Supplementary information

Table S2. Standard curve parameters (expression and r^2), and experimental Ct values (mean, s.e.m., minimum and maximum) for all assays

Gene	Standard Curve		Ct Values			
	Efficiency (%)	r^2	Mean	s.e.m.	Minimum	Maximum
18s	*996*	*0.671*	*5.34*	*0.05*	*4.45*	*7.03*
β-Actin	*117*	*0.988*	*3.41*	*0.03*	*2.50*	*4.17*
**CDCC84**	**100**	**0.994**	**10.08**	**0.03**	**9.34**	**11.01**
DnaJA2	*103*	*0.993*	*6.32*	*0.05*	*5.05*	*8.62*
EF1-α	*117*	*0.990*	*3.80*	*0.02*	*3.38*	*4.32*
External Standard	*142*	*0.952*	*9.66*	*0.09*	*7.31*	*12.45*
MrpL40	*103*	*0.996*	*7.31*	*0.05*	*5.94*	*8.54*
**SEP15**	**106**	**0.998**	**9.94**	**0.03**	**9.05**	**10.84**
HCN1	110	0.993	13.42	0.22	9.02	18.03
HCN2a1	107	0.995	11.62	0.11	9.35	14.48
HCN2a2	113	0.993	13.00	0.16	10.61	17.36
HCN2b1	112	0.995	14.30	0.12	10.74	17.80
HCN2b1/2	111	0.993	12.59	0.12	9.90	15.95
HCN3	109	0.993	15.20	0.24	10.89	22.20
HCN4a1/2	101	0.998	11.04	0.21	7.93	16.03
HCN4b	*111*	*0.913*	*18.24*	*0.26*	*11.80*	*23.05*
$Ca_v1.3$	110	0.989	13.93	0.11	10.82	16.52
$Ca_vβ2$	111	0.994	10.20	0.04	8.71	11.61
calsequestrin	117	0.990	4.07	0.03	3.20	5.06
NCX	122	0.987	4.04	0.03	3.37	5.18

Journal of Experimental Biology • Supplementary information

RYR3	99	0.998	9.76	0.17	6.31	13.62
SERCA2	149	0.973	15.52	0.05	14.42	17.30
S100	106	0.993	7.83	0.04	6.74	9.05
TRPC1	122	0.993	16.06	0.18	12.17	20.87
ERG	110	0.994	4.38	0.04	3.56	5.67
$K_{ir}2.1$	109	0.985	12.66	0.05	11.55	14.77
$K_{ir}2.2$	102	0.990	13.23	0.10	10.07	16.33
$Na_v1.4$	116	0.991	6.27	0.03	5.56	7.61
$Na_v1.5$	108	0.994	8.47	0.08	7.08	11.28
$Na_v1.6$	114	0.976	16.93	0.26	11.38	22.98
NKA α1a	127	0.989	15.77	0.14	12.12	21.28
NKA α1b	*133*	*0.771*	*17.36*	*0.07*	*13.58*	19.63
NKA α1c	102	0.991	5.82	0.04	4.78	7.72
NKA α3	114	0.992	3.96	0.03	3.31	5.01
α_{1A} Adrenoceptor	*164*	*0.954*	*15.84*	*0.11*	*11.39*	*18.84*
α_{1B} *Adrenoceptor*	112	0.986	3.06	0.03	2.50	3.86
β_2 Adrenoceptor	*88*	*0.930*	*16.56*	*0.10*	*14.08*	*19.67*
ANP	*132*	*0.979*	*13.71*	*0.17*	*10.33*	*20.97*
col1α1	113	0.996	5.19	0.07	3.57	6.76
Cx43	114	0.998	10.74	0.07	8.71	13.16
TGF-β1	109	0.990	10.68	0.06	8.58	12.54

Target genes analysed are neither bold nor italics, reference genes used for analysis are in bold, and genes excluded from analysis are in italics.

Journal of Experimental Biology • Supplementary information

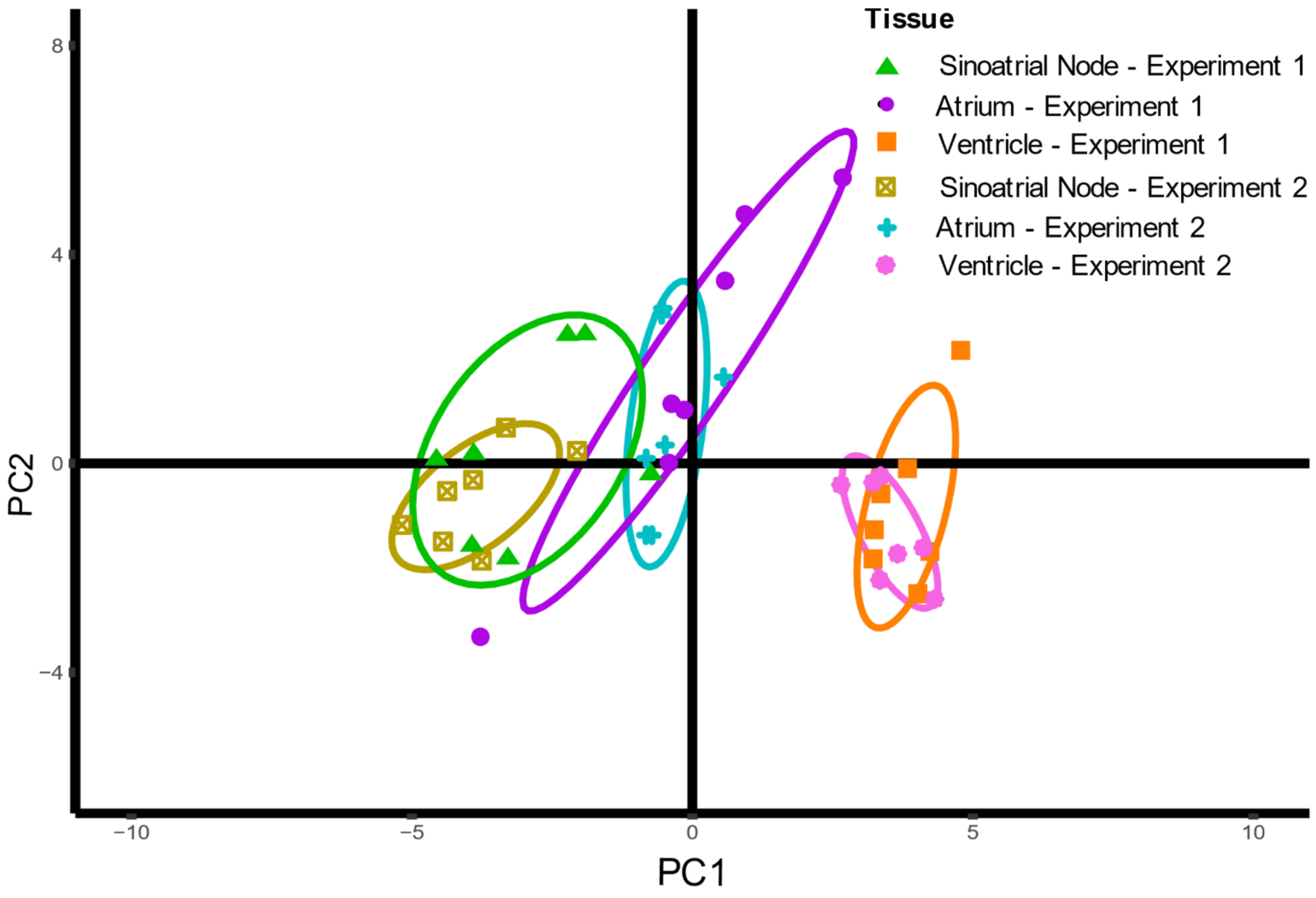

Fig. S2. Principal component analysis (PCA) of mRNA expression of cardiac function genes. A comparison of tissue types from fish in experiment 1 and experiment 2 acclimated for more than 3 weeks. All $E^{-\Delta\Delta Ct}$ (expression normalized to reference genes and interrun calibratior (IRC)) data shown in Fig. 3 and similar data from experiment 2 were used for this PCA analysis for sinoatrial node (SAN), atrial and ventricular mRNA expression of fish acclimated to 4°C for more than 3 weeks in experiment 1 and 2 (n=7). PC1 and PC2 explained 34.9% and 14.3% of variance, respectively. Each tissue type and experimental group is identified by a different symbol and a 68% confidence limit ellipse.

Table S3. Loading factor components.

Loading Factors	Figure 2A		Figure 2B		Figure 2C		Figure 2D		Figure S2	
	PC1	PC2	PC1	PC2	PC1	PC2	PC1	PC2	PC1	PC2
HCN1	-29%	33%	33%	17%	-24%	-31%	24%	16%	-24%	-9%
HCN2a1	-22%	15%	15%	-15%	-25%	5%	34%	1%	-27%	9%
HCN2a2	-10%	4%	4%	-23%	-18%	19%	30%	-9%	-22%	5%
HCN2b1	-6%	32%	32%	13%	-22%	-30%	26%	-1%	-12%	19%
HCN2b1/2	-15%	30%	30%	-7%	-29%	-12%	33%	-9%	-19%	24%
HCN3	-29%	13%	13%	7%	0%	-12%	-15%	-14%	-30%	2%
HCN4a1/2	-28%	7%	7%	19%	4%	-19%	6%	30%	-28%	10%
$Ca_v1.3$	-28%	30%	30%	9%	-19%	-25%	24%	22%	-25%	-10%
$Ca_v\beta2$	-25%	-2%	-2%	8%	-14%	-1%	4%	22%	-22%	-22%
calsequestrin	0%	14%	14%	-31%	-28%	21%	25%	-11%	-2%	13%
NCX	12%	13%	13%	-31%	-26%	19%	16%	-31%	18%	9%
RYR3	21%	13%	13%	-26%	-13%	21%	3%	-24%	25%	-5%
SERCA2	13%	5%	5%	-36%	-21%	29%	12%	-35%	11%	26%
s100	-8%	6%	6%	-24%	-17%	23%	-11%	-18%	-7%	-29%
TRPC1	-29%	31%	31%	15%	-26%	-28%	28%	17%	-27%	-14%
ERG	-5%	9%	9%	6%	-10%	2%	-5%	3%	-14%	29%
$K_{ir}2.1$	9%	-6%	-6%	-9%	-8%	15%	-11%	-13%	9%	-33%
$K_{ir}2.2$	-18%	-18%	-18%	6%	7%	5%	-15%	5%	-18%	-10%
$Na_v1.4$	2%	28%	28%	-16%	-29%	3%	11%	-33%	7%	26%
$Na_v1.5$	-15%	1%	1%	13%	7%	-2%	-17%	-3%	-21%	26%
$Na_v1.6$	-29%	24%	24%	26%	-15%	-30%	1%	32%	-14%	-19%

Journal of Experimental Biology • Supplementary information

NKA α1a	-12%	2%	2%	-26%	-15%	21%	0%	3%	-13%	2%
NKA α1c	15%	6%	6%	-38%	-23%	30%	8%	-36%	15%	-17%
NKA α3	17%	25%	25%	-3%	-20%	-3%	1%	-12%	24%	-4%
$\alpha_{1\beta}$ *Adrenoceptor*	-23%	4%	4%	-1%	-11%	1%	20%	10%	-6%	-29%
col1α1	-24%	31%	31%	9%	-22%	-19%	17%	6%	-18%	-3%
Cx43	-14%	1%	1%	-2%	-8%	4%	-14%	10%	-4%	-35%
TGF-β1	-13%	-26%	-26%	1%	17%	15%	-33%	-3%	-19%	-9%
Total proportion of variance	36%	24%	24%	22%	24%	18%	27%	20%	35%	14%
Standard deviation	3.17	2.59	2.59	2.49	2.57	2.22	2.77	2.35	3.12	2.00

Journal of Experimental Biology • Supplementary information

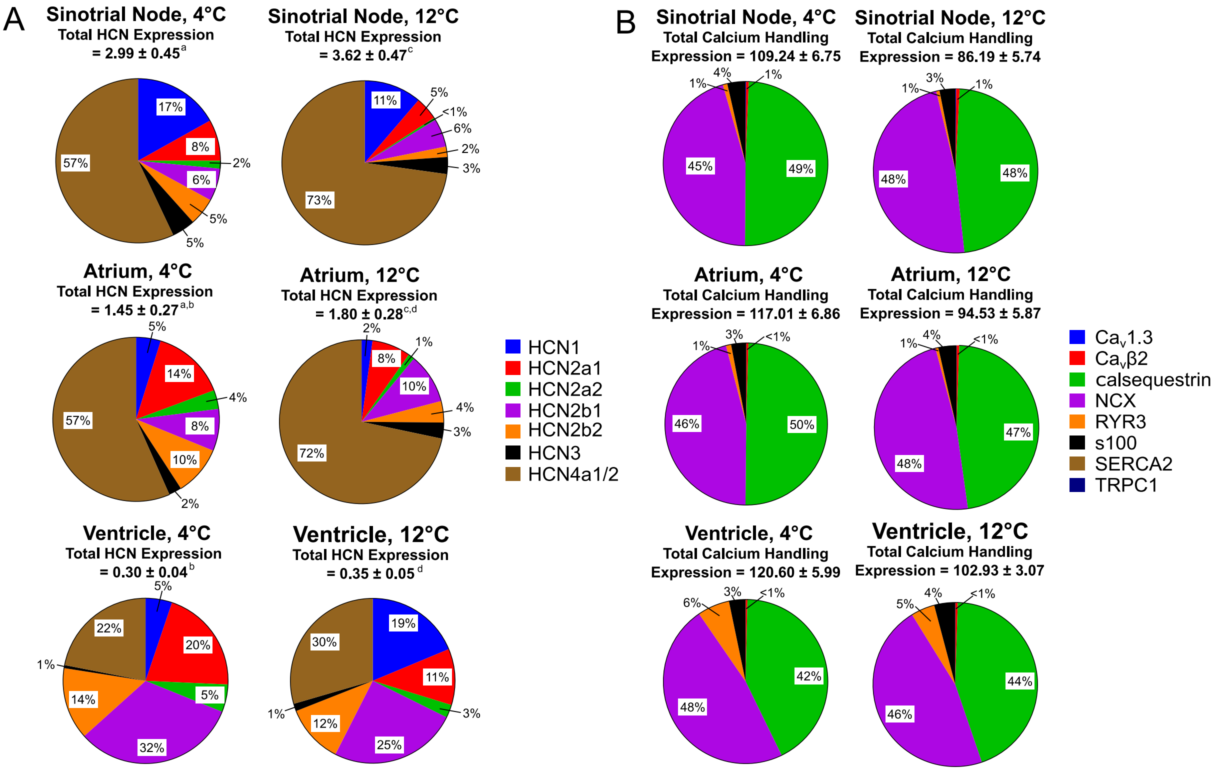

Fig. S3 - Relative mRNA expression of proteins hypothesized to play a role in pacemaker cell spontaneous depolarization in SAN, atrial and ventricle tissue from fish acclimated at 4°C and 12°C for more than 3 weeks in experiment 1.(A) Relative mRNA expression of different HCN channels. Total HCN expression is the expression ($E^{-\Delta Ct}$) normalized to the geometric mean of the reference genes (CCDC84 and SEP15) of all genes in the chart. HCN2b2 was calculated from the difference in expression of HCN2b1/2 and HCN2b1. (B) Relative mRNA expression of different calcium handling proteins. Total calcium handling expression is the expression ($E^{-\Delta Ct}$) normalized to the geometric mean of the reference genes (CCDC84 and SEP15) of all genes in the chart. Expression of *Ca$_v$1.3*, *SERCA2* and *TRPC1* are <1% of total calcium handling expression for all tissues and temperatures and are not labelled on the pie charts. Dissimilar letters are used to indicate statistically significant differences in total expression between tissues within a thermal acclimation temperature (one-way ANOVA) ($P<0.05$).

Journal of Experimental Biology • Supplementary information

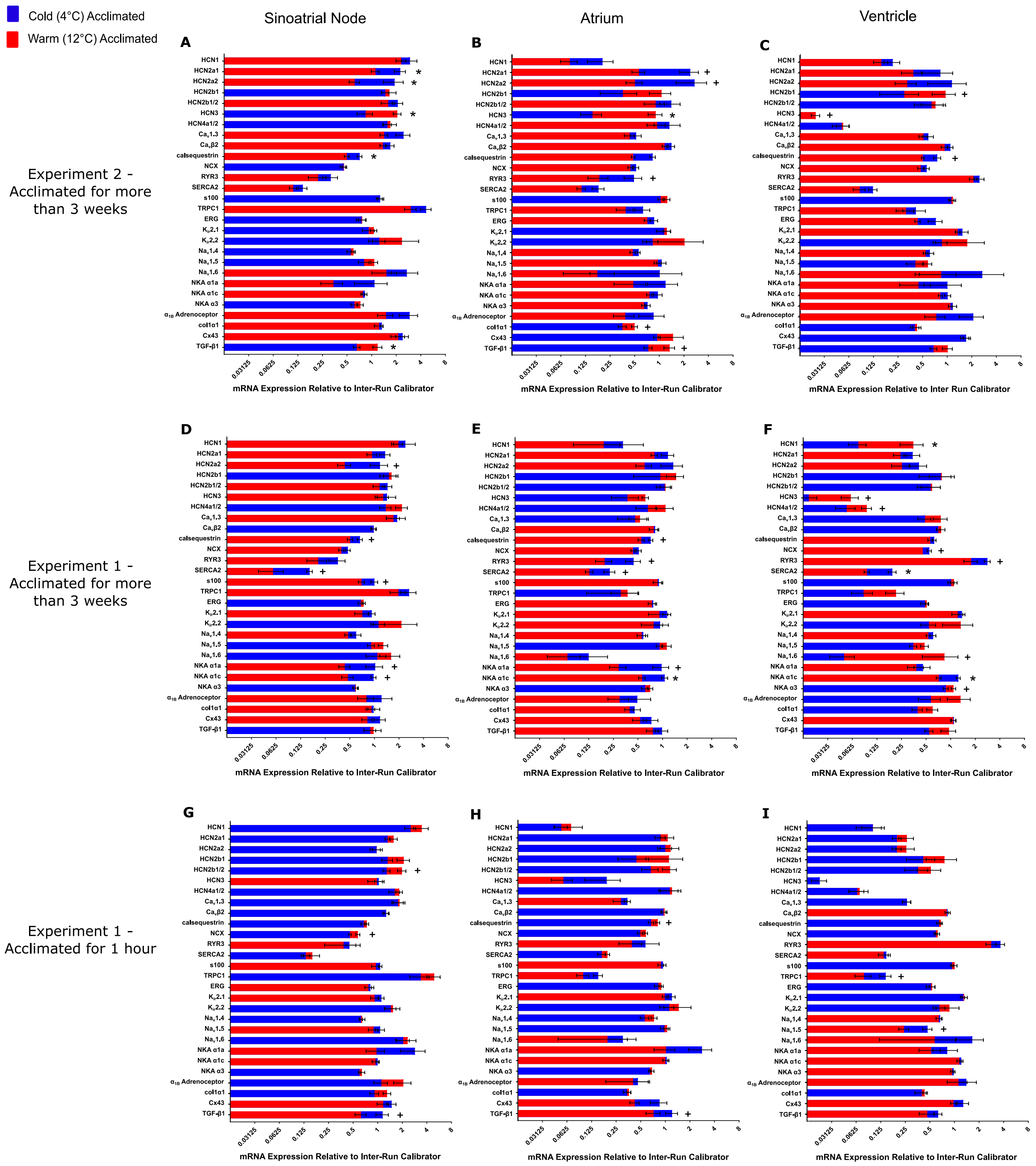

Fig. S4 – mRNA expression of cardiac function genes. (A) SAN, (B) atrium, and (C) ventricle of fish acclimated to 4°C and 12°C for more than 3 weeks for fish in experiment 1; the (D) SAN, (E) atrium and (F) ventricle of fish acclimated to 4°C and 12°C for more than 3 weeks in experiment 2; and the (G) SAN, (H) atrium and (I) ventricle of fish acclimated to 4°C and 12°C for 1 hour with an intrinsic heart rate resetting response. Expression ($E^{-\Delta\Delta Ct}$) was normalized to the geometric mean of the expression of reference genes (*CCDC84* and *SEP15)*, and then to an inter-run calibrator, and values are presented as means±s.e.m (n=7 except in those examples specified in the methods). * Statistically significant differences after FDR adjustment and + statistically significant differences without FDR adjustment (P<0.05) between cold and warm acclimated fish.

Journal of Experimental Biology • Supplementary information

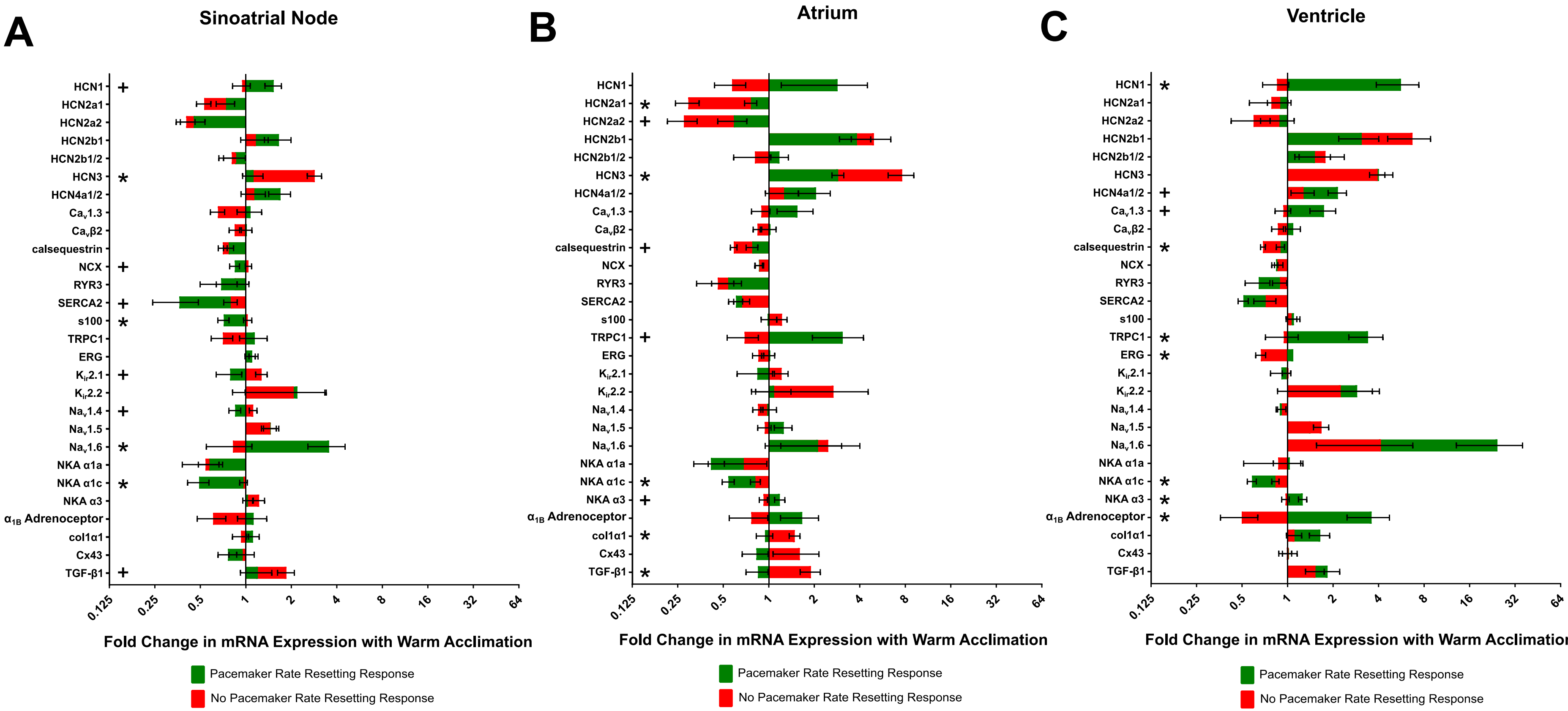

Figure S5 - Fold change in mRNA expression of cardiac function genes from fish acclimated to 4°C and 12°C for more than 3 weeks that reset intrinsic heart rate (experiment 1) and didn't reset intrinsic heart rate (experiment 2). (A) Sinoatrial node, (B) atrial and (C) ventricle tissues. Expression (fold change in $E^{-\Delta\Delta Ct}$) was normalized to the geometric mean of the expression of the reference genes (*CCDC84* and *SEP15)*, an inter-run calibrator, and then to control cold (4°C) acclimated fish and values are presented as means±s.e.m. (n=7) . * Statistically significant differences after FDR adjustment and + statistically significant differences without FDR adjustment (<0.05) between intrinsic heart rate resetting responses (Student's t test or Mann-Whitney test).
